# Detection of Dengue Virus among Children with Suspected Malaria, Accra, Ghana

**DOI:** 10.3201/eid2408.180341

**Published:** 2018-08

**Authors:** Nicholas Amoako, Samuel Duodu, Francis E. Dennis, Joseph H.K. Bonney, Kwaku P. Asante, Juliana Ameh, Lydia Mosi, Takaya Hayashi, Eudosia E. Agbosu, Deborah Pratt, Darwin J. Operario, Barry Fields, Jie Liu, Eric R. Houpt, George E. Armah, Justin Stoler, Gordon A. Awandare

**Affiliations:** University of Ghana, Legon, Ghana (N. Amoako, S. Duodu, F.E. Dennis, J.H.K. Bonney, L. Mosi, T. Hayashi, E.E. Agbosu, D. Pratt, G.E. Armah, G.A. Awandare);; Kintampo Health Research Centre, Kintampo, Ghana (N. Amoako, K.P. Asante);; Ledzokuku Krowor Municipal Assembly Hospital, Teshie, Accra, Ghana (J. Ameh);; Tokyo Medical and Dental University, Tokyo, Japan (T. Hayashi);; University of Virginia, Charlottesville, Virginia, USA (D.J. Operario, J. Liu, E.R. Houpt);; Centers for Disease Control and Prevention, Atlanta, Georgia, USA (B. Fields);; University of Miami, Florida, USA (J. Stoler).

**Keywords:** Dengue, malaria, febrile illness, children, DNV2, TaqMan array card, multi-pathogen, assay, Accra, Kintampo, Ghana, Plasmodium, malaria parasitemia, co-infected, West Africa, endemic, viruses

## Abstract

We report new molecular evidence of locally acquired dengue virus infections in Ghana. We detected dengue viral RNA among children with suspected malaria by using a multipathogen real-time PCR. Subsequent sequence analysis revealed a close relationship with dengue virus serotype 2, which was implicated in a 2016 outbreak in Burkina Faso.

The accurate diagnosis of nonmalarial febrile illnesses remains a large challenge in many malaria-endemic countries (*1*). The etiologic agents in this context are often not identified because of nonspecific clinical symptoms and diagnostic limitations (*2*); for example, of 457 patients in Nigeria who were presumptively treated for malaria, only 3.9% tested positive (*3*). Because of the decline in malaria transmission over the past decade in many endemic areas, including Ghana, there is a critical need for a comprehensive characterization of the etiology of acute febrile illness (AFI) (*4*). Dengue virus infections cause symptoms that are similar to those of malaria, and considering the increasing reports of dengue outbreaks in countries neighboring Ghana (*5**–**8*), there is an increased need for dengue surveillance.

## The Study

To obtain a description of the pathogens causing febrile illnesses in Ghana, we conducted a hospital-based cross-sectional study among children in 2 geographically distinct areas. Kintampo, in the Brong Ahafo region, is a semiurban area in the forest savanna middle belt and has a population of 42,957. Teshie is a periurban area in the Greater Accra region that has ≈171,875 residents (Technical Appendix Figure). We conducted the study during October 2016–July 2017, encompassing parts of the dry season (November–March) and rainy season (April –July). Children 1–15 years of age whose symptoms included fever were examined in the outpatient departments of Kintampo Municipal Hospital or Ledzokuku Krowor Municipal Assembly Hospital in Teshie. The study was approved by the ethics review committees of the Noguchi Memorial Institute for Medical Research, University of Ghana, the Ghana Health Service, and the Kintampo Health Research Centre. We recruited the patients for this study after we obtained written informed consent from their parents or guardians. The inclusion criteria were fever within the preceding 24 hours or measured axillary temperature ≥38°C occurring for <7 days and no severe or known chronic disease. Attending clinicians at the 2 sites screened a total of 10,234 children, and 700 were enrolled for the study on the basis of the inclusion criteria. We collected venous blood (5 mL) from each participant into EDTA-containing tubes (BD Vacutainer; Becton Dickinson, Franklin Lakes, NJ, USA) for malaria tests and full blood counts; the remaining blood was stored at −80°C until use. All of the children were treated according to the Ghana Health Service treatment guidelines.

We randomly selected stored blood samples from 166 children diagnosed with AFI and screened the samples by using a customized multipathogen, real-time PCR–based TaqMan probe-array card (TAC; Applied Biosystems, Carlsbad, CA, USA), as described by Liu et al. (*9*). The AFI TAC assay simultaneously tests for 26 pathogens, including 3 protozoa, 7 bacteria, and 16 viruses (Table 1). Each card tests 6 samples and 2 controls (*9*).

**Table 1:** Pathogens tested for by using the customized AFI TaqMan array card used in study of dengue virus among 166 children with suspected malaria, Accra, Ghana, October 2016–July 2017*

Pathogens
*Bartonella* spp.
*Brucella* spp.
Bundibugyo virus
*Coxiella burnetii*
Crimean-Congo hemorrhagic fever virus
Chikungunya virus
Dengue virus
Ebola virus
Hepatitis E virus
HIV
Lassa virus
*Leishmania* spp.
*Leptospira* spp.
Marburg virus
Nipah virus
O’nyong-nyong virus
*Plasmodium* spp.
*Rickettsia* spp.
Rift Valley fever virus
*Salmonella* spp.
Sudan virus
*Trypanosoma brucei*
West Nile virus
*Yersinia pestis*
Yellow fever virus
Zika virus

*Applied Biosystems, Carlsbad, CA, USA, AFI, acute febrile illness.

**Figure 1:**
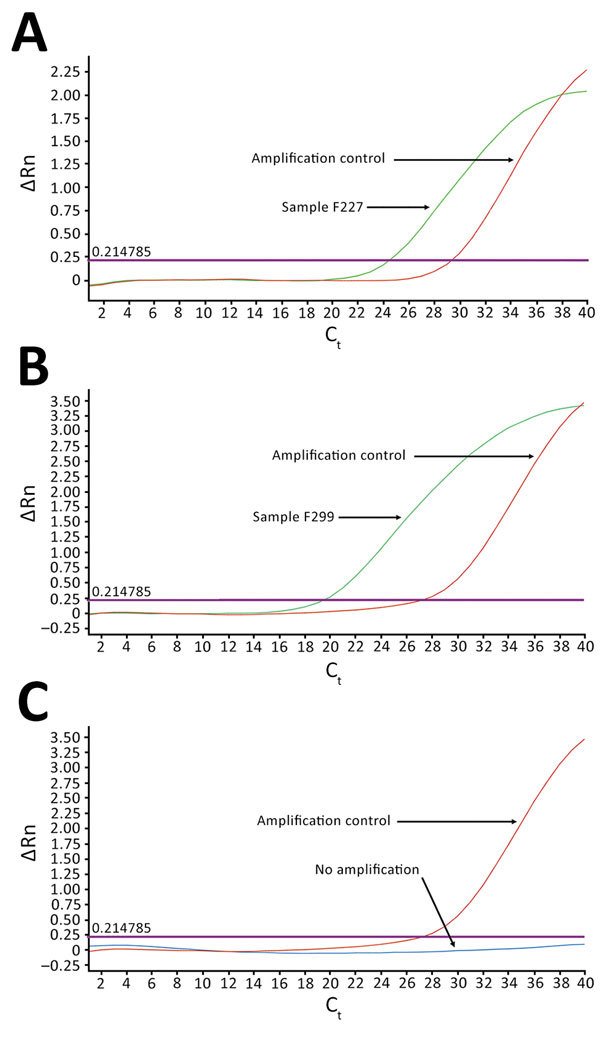
TaqMan array card amplification plots for 2 dengue virus–positive samples and 1 negative sample in study of dengue virus among 166 children with suspected malaria, Accra, Ghana, October 2016–July 2017. Blood samples (2.5 mL of the 5.0 mL collected) obtained from children reporting to the hospital with acute febrile illness (AFI) were screened for 26 pathogens simultaneously by using the real-time PCR TaqMan array card. The cards were in 384-well format, and each well contained 1 µL of reaction mixture (0.75 µL of the extracted total nucleic acid and 0.25 µL of TaqMan Fast Virus 1-step Master Mix; Life Technologies/Applied Biosystems, Foster City, CA, USA). The assays were run on an Applied Biosystems Quant Studio 7 Flex real-time PCR system, according to the manufacturer’s recommendations. Two samples tested positive for dengue virus: A) F227, which amplified with critical C_t_ of 24.40; and B) F299, which amplified with C_t_ of 19.35. C) All others tested negative, indicated by amplification signals (ΔRn) below threshold levels at quantification cycle cutoff of 35. Each assay included nucleic acid to serve as amplification controls. External controls were spiked into each to monitor extraction and amplification efficiency, and 1 negative control was included for each batch of extraction to monitor laboratory contamination. C_t_, cycle threshold.

As we expected, *Plasmodium* spp. was the predominant pathogen detected in samples from the children (36.8% of samples tested; Table 2). *Salmonella enterica* serovar Typhi, *Rickettsia* spp., *Coxiella burnetii*, and HIV-1 were also detected in some samples (Table 2). However, the most notable observation was the detection of dengue virus in samples from 2 children, 3 and 14 years of age, who were admitted to Ledzokuku Krowor Municipal Assembly Hospital; critical cycle threshold (C_t_) for these children was 24.40 and 19.35, respectively (Figure 1). Furthermore, 1 of the dengue-positive samples was also positive for *Plasmodium* spp., providing a vivid demonstration of the complex etiology of AFI in malaria-endemic areas. This observation is consistent with a recent report showing that 51% of febrile children in Ghana who were diagnosed with malaria parasitemia were co-infected with >1 pathogen (*10*).

**Table 2:** Pathogens detected in study of dengue virus among 166 children with suspected malaria by using customized AFI TaqMan array card, Accra, Ghana, October 2016–July 2017*

Pathogen(s)	No. (%) patients
*Plasmodium* spp. only	61 (36.8)
Dengue virus only	1 (0.6)
*Plasmodium* spp. + dengue virus	1 (0.6)
*Salmonella enterica* serovar Typhi	1 (0.6)
*Rickettsia* spp.	5 (3.0)
*Coxiella burnetii* + *Plasmodium* spp.	1 (0.6)
HIV	1 (0.6)
Unidentified (negative result)	95 (57.2)

*AFI, acute febrile illness.

**Figure 2:**
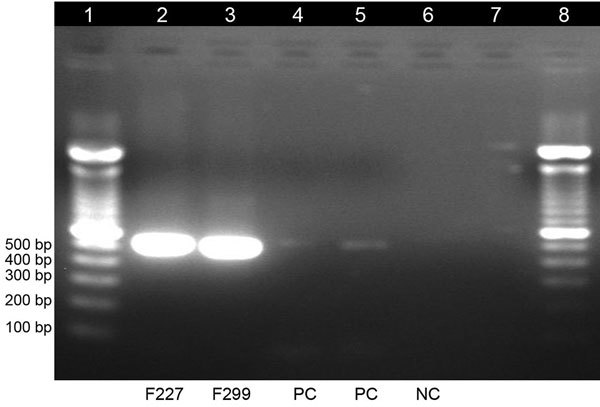
Gel electrophoresis of dengue virus–specific RT-PCR products in study of dengue virus among 166 children with suspected malaria, Accra, Ghana, October 2016–July 2017. We completed a conventional RT-PCR assay by using dengue-specific primers from Lanciotti et al. (*15*) to confirm the results of the TaqMan array card assays. The amplification products (expected size 511 bp) were electrophoresed on 2% agarose gel, stained with ethidium bromide, and viewed under ultraviolet light. Lane 1, molecular weight marker; lanes 2 and 3, test samples; lanes 4 and 5, positive controls; lane 6, negative control; lane 7, empty; lane 8, molecular weight marker. RT-PCR, reverse transcription PCR.

No dengue virus infections have previously been reported in Ghana, despite suspected transmission after the isolation of dengue virus type 2 (DENV-2) in travelers from Finland who visited Ghana during 2000–2005 (*11*) and our previous study showing serologic evidence of prior dengue exposure among malaria-positive children in Ghana during 2011–2014 (*12*). Therefore, we sought to confirm the results of the TAC screening by using 3 additional molecular methods: 1) the Trioplex TaqMan-based real-time reverse transcription PCR (RT-PCR) developed by the Centers for Disease Control and Prevention (*13*), which differentiates dengue from the arboviruses chikungunya (CHIKV) and Zika; 2) a second TaqMan-based real-time RT-PCR assay developed by Johnson et al. for dengue virus serotype detection (*14*); and 3) a conventional RT-PCR assay by using primers as described by Lanciotti et al. (*15*) (Figure 2). All of these methods confirmed the samples to be dengue positive. C_t_ values for DENV obtained from the Trioplex assay for the 2 samples were 26.96 and 34.70; results for CHIKV and Zika virus were undetermined (negative). We recorded C_t_ values of 27.53 and 35.42 on the Johnson et al. real-time RT-PCR assay, which also characterized the 2 samples as DENV-2. 

We further sequenced viral RNA by using an ABI 3130XL Genetic Analyzer (Applied Biosystems, Carlsbad, CA, USA). BLAST search (https://blast.ncbi.nlm.nih.gov/blast.cgi) using default settings of generated nucleotide sequences revealed that the viruses in both positive samples were identical (100% homology) and closely related to the DENV-2 strains isolated in the 2016 outbreak in Burkina Faso (GenBank accession no. KY627763.1). We deposited the nucleotide sequences of the detected virus strain in GenBank under accession nos. MG937762 and MG937763. 

Apart from fever, both dengue-positive children reported chills, cough, and vomiting; however, neither reported diarrhea or rash. On a follow-up visit 2 months after enrollment, the children from whom we obtained the dengue-positive samples seemed healthy and afebrile, suggesting that no severe complications of the infection developed. Convalescent-phase blood samples from both patients tested positive for dengue-specific IgG; 1 was additionally positive for dengue IgM (Abcam human anti-dengue virus IgG and IgM ELISA kit; Abcam, Cambridge, UK). The parents reported that these children had not traveled outside the country, indicating that the infection was locally acquired.

## Conclusions

Our previous investigations detected dengue antibodies in 21.6% of children in 3 areas of Ghana, including Kintampo and Accra; however, no virus was detected from any of them, suggesting previous exposure rather than acute infections (*12*). In this study, we have now confirmed the presence of dengue virus in the blood of two children, indicating acute infections. In light of other reports from elsewhere in West Africa (*5**–**7*), this DENV-2 strain may soon become regionally endemic, if it has not already. We have advised the Ghana Health Service accordingly to take measures to intensify surveillance, consistent with prior recommendations (*8*).

## Acknowledgments

Technical Appendix Map of Ghana showing hospital study sites of Teshie and Kintampo in study of dengue virus among 166 children with suspected malaria, Accra, Ghana, October 2016–July 2017.
