# Supplementary material for: Detection of Dengue Virus among Children with Suspected Malaria, Accra, Ghana
Source: Emerg Infect Dis. 2018 Aug;24(8):1544–7. doi: 10.3201/eid2408.180341 (PMC6056106; doi:10.3201/eid2408.180341)
Supplement: Technical Appendix — Map of Ghana showing hospital study sites of Teshie and Kintampo in study of dengue virus among 166 children with suspected malaria, Accra, Ghana, October 2016–July 2017. [file 18-0341-Techapp-s1.pdf]

Article DOI: https://doi.org/10.3201/eid2408.180341

Detection of Dengue Virus Detected among Children with Suspected Malaria by using a Multiple-pathogen Assay, Accra, Ghana

Technical Appendix

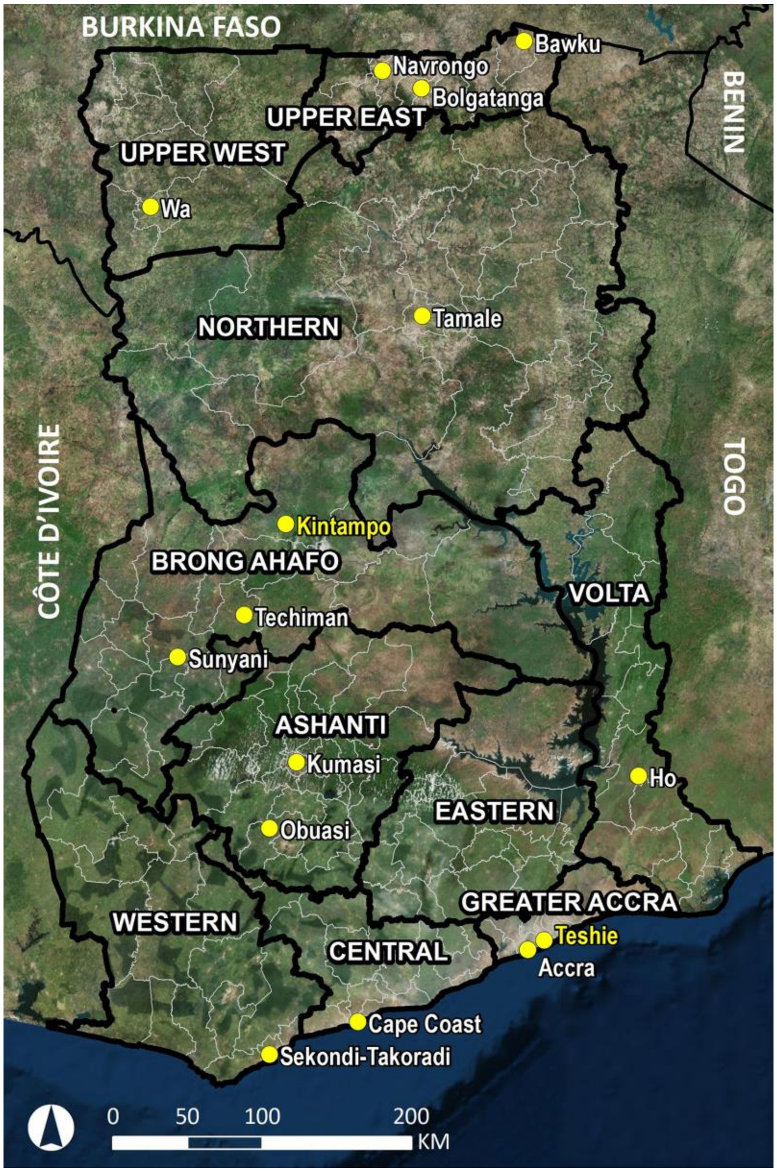

Technical Appendix Figure. Map of the Republic of Ghana. Yellow dots indicate cities and towns; hospital study sites of Teshie and Kintampo are indicated in yellow text. Regions are in all capital letters.
